# Plasma Cell Myeloma Within a Renal Cell Carcinoma, an Intimate Histologic Relationship: A Case Report and Literature Review

**DOI:** 10.7759/cureus.12898

**Published:** 2021-01-25

**Authors:** Lee B Syler, Casey Gooden, Nicole Riddle

**Affiliations:** 1 Pathology, University of South Florida, Tampa, USA; 2 Pathology, Ruffolo, Hooper & Associates, Tampa, USA

**Keywords:** renal cell carcinoma, clear cell renal cell carcinoma, plasma cell myeloma, multiple myeloma, renal cell carcinoma and plasma cell myeloma, synchronous tumors

## Abstract

The coexistence of two separate malignancies in a patient is a rare occurrence. Even more infrequent is the coexistence of a hematologic malignancy and a solid tumor. However, the relationship between renal cell carcinoma (RCC) and plasma cell myeloma (PCM) has been reported in previous studies. These studies described synchronous cases of RCC and PCM and demonstrated that this situation occurs more frequently than expected by probability calculations.

We present, what we believe to be, the first reported case of RCC directly and physically involved by PCM and, we review the literature on the association between these malignancies and explore possible mechanisms for their higher than expected association. In describing this case, emphasis is made to describe unique histologic findings that could further support a more direct and intimate association between these tumors.

## Introduction

Plasma cell myeloma (PCM) is a malignant neoplastic proliferation of plasma cells. It is usually associated with an M protein spike in serum and/or urine, and end-organ damage. The bone marrow is the site of origin of almost all PCM and most cases have disseminated bone marrow involvement. Other organs can be secondarily involved [1].

Renal cell carcinoma (RCC) is a malignant neoplasm derived from renal epithelial cells. Its most common variant, clear cell renal cell carcinoma (CCRCC) accounts for approximately 60% of this group [2]. Genetically, it is most characterized by deletions of the short arm of chromosome 3 and mutations of the VHL gene. In most cases, it is asymptomatic and discovered incidentally on imaging for other symptoms. Treatment is most commonly through nephrectomy or partial nephrectomy [2].

The simultaneous occurrence of two malignancies in a patient is a rare event. Yet, several studies have reported on the relationship between RCC and PCM [3-9]. These studies show a higher than expected concomitant incidence between them. Many different hypotheses have been proposed to explain the mechanisms behind this association [3,5,6], but none have been clearly proven to date. Some include shared risk factors such as age, lifestyle, environmental exposures, and genetic mutations. Others include treatment effects of the first cancer and certain cytokines.

## Case presentation

**Figure 1:**
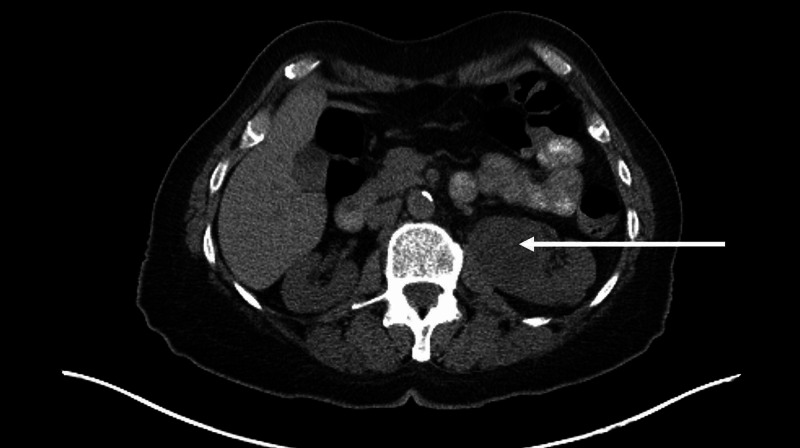
Outside imaging of left renal mass (CT scan) Arrow: Indicates the left renal mass

**Figure 2:**
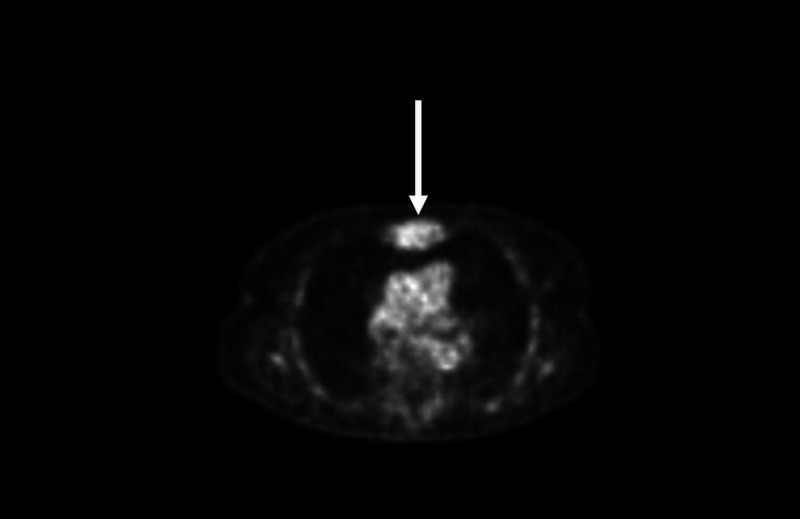
Outside imaging of sternal plasmacytoma (PET Scan) Arrow: Indicates PET avid sternal plasmacytoma PET: Positron emission tomography

A 68-year-old female was referred to us from an outside institution for the evaluation and surgical removal of a left renal mass. The patient had an eight-year history of IgA, kappa light chain PCM in multiple sites which included multiple lytic bone lesions, a sternal lesion, and a right chest wall lesion. Outside imaging was submitted for review (Figures 1 and 2). She had been treated with multiple lines of systemic chemotherapy as well as radiation therapy. She had been on lenalidomide and received radiation therapy to the chest just before her referral. She suffered from chronic cytopenia which was treated with epoetin alfa (Procrit), romiplostim (Nplate), and filgrastim (Neupogen).

She was admitted for a left robotic nephrectomy. During the procedure, she was found to have a periaortic mass, that had not been visualized on the submitted outside imaging. The mass was intricately adhered to the hilum and was extremely difficult to separate from the kidney. After the nephrectomy, the surgery was converted to an open procedure for the debulking of the periaortic mass. The total operative time and total blood loss were six hours and 100 ml, respectively.

**Figure 3:**
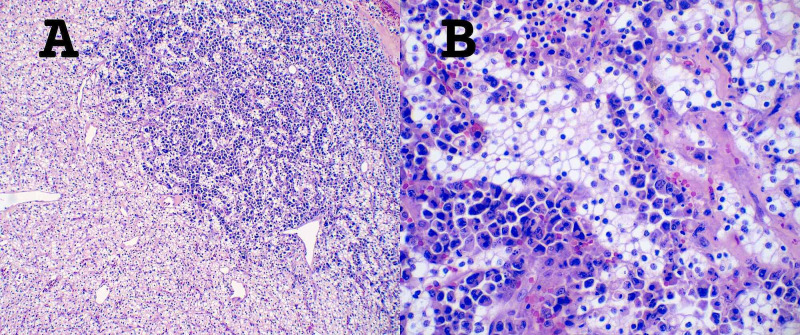
Tumor-to-tumor interface *A.* HE stain, 10x magnification. Clear cell renal cell carcinoma invaded by plasma cell myeloma. *B.* HE stain, 40x magnification. Shows a higher power view of the infiltrative pattern HE: Hematoxylin and eosin

On gross examination, the left nephrectomy specimen revealed a 5.0 x 4.8 x 4.0 cm mass within the renal sinus, which was golden-yellow, soft, bulging, well-demarcated, and with central hemorrhage. Upon microscopic examination, there were monomorphic cells in acinar patterns with clear cytoplasm and an intricate network of capillary vasculature, consistent with CCRCC, nuclear grade 2. The tumor was limited to the kidney. The most impressive finding was that the CCRCC had variegated areas of infiltrating plasmacytoid cells with nuclear pleomorphism, hyperchromatic nuclei, and clumped chromatin, consistent with the patient’s history of PCM. Interestingly, at the tumor-to-tumor interface areas, nests of the two populations seemed to intermix without a clear boundary (Figure 3).

**Figure 4:**
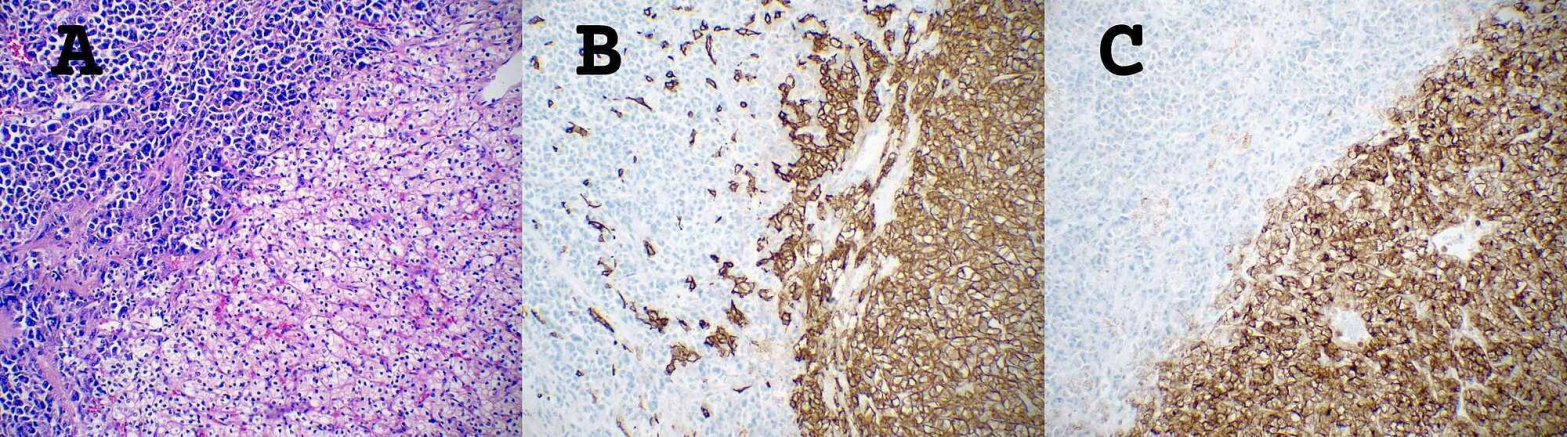
Immunohistochemistry for clear cell renal cell carcinoma component *A. *HE stain, 20x magnification. CCRCC at the bottom right half and PCM at the upper left half of the image, showing the tumor-to-tumor border. *B. *Positive CA-IX immunostaining of the CCRCC component, 20x magnification. *C.* Positive CD10 immunostaining of the CCRCC component, 20x magnification CCRCC: Clear cell renal cell carcinoma; HE: Hematoxylin and eosin; PCM: Plasma cell myeloma

Immunohistochemistry of the neoplastic cells was positive for CA-IX and CD10 and negative for CK7 (Figure 4).

**Figure 5:**
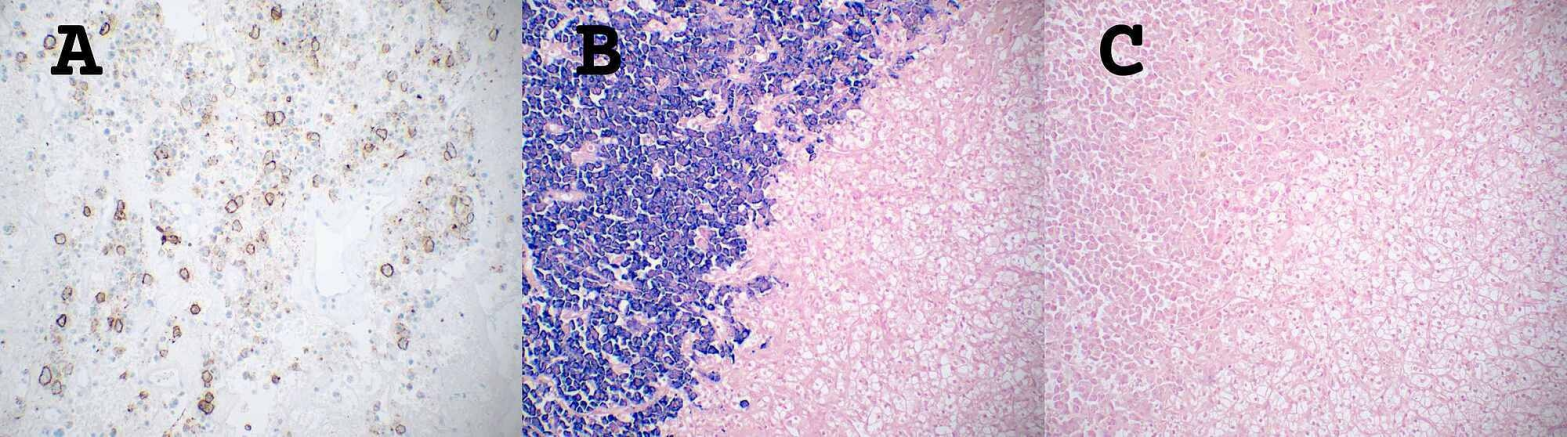
Immunohistochemistry for plasma cell myeloma component *A. *Positive immunostaining for CD138, 20x magnification. *B. *Positive ISH for kappa light chain, 20x magnification. *C.*  Negative ISH for lambda light chain, 20x magnification ISH: In situ hybridization

The scattered areas of PCM within the CCRCC were positive for CD138 and kappa light chain, and negative for lambda light chain, CA-IX, and CD10 (Figure 5).

**Figure 6:**
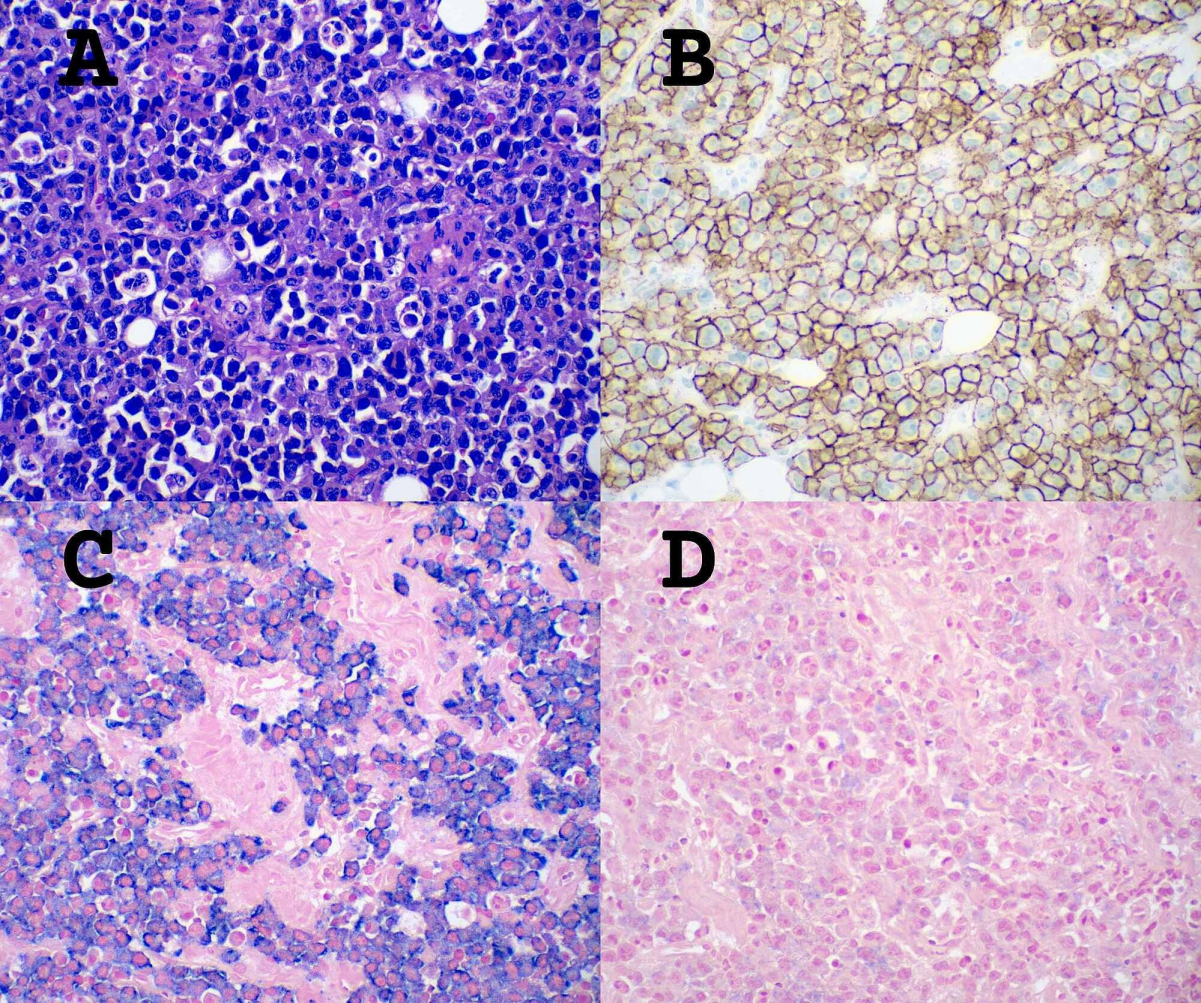
Immunohistochemistry of para-aortic mass *A. *HE stain, 40x magnification, of diffuse proliferation of neoplastic plasma cells. *B.* Positive immunostaining for CD138, 40x magnification. *C.* Positive ISH for Kappa light chain, 40x magnification. *D. *Negative ISH for Lambda light chain, 40x magnification HE: Hematoxylin and eosin; ISH: In situ hybridization

The excised para-aortic mass was 5.1 x 4.6 x 3.0 cm, tan-red, and had a rubbery consistency. Microscopic examination showed diffuse proliferation of plasma cells involving fibro-adipose tissue, skeletal muscle, and a large vessel. These plasma cells had the same morphologic features and stained positive for the same plasma cell markers as the previous infiltrating plasma cell component of the renal mass, consistent with PCM (Figure 6).

The patient had a total hospital stay of three days without postoperative complications. She was later discharged to home for continued outpatient management. However, per clinical notes, she continued management at an outside facility with palliative radiation therapy, chemotherapy, and platelet count monitoring. No further information was available on her current disease status.

## Discussion

PCM accounts for approximately 1% of malignancies and 10-15% of hematopoietic neoplasms [1]. In 2015, an estimated 26,000 new cases were diagnosed in the US [1]. It is slightly more common in men than in women (1.1/1) and its incidence increases progressively with age; with about 90% of cases occurring in patients >50 years of age [1].

RCC accounts for approximately 2% of malignancies [2]. In 2013, an estimated 51,200 renal and pelvic cancers were diagnosed in the US [2]. It is two times more frequent in men than in women and the peak incidence occurs between the fifth and sixth decade of life [2].

Throughout the literature concerning the relationship between RCC and PCM, evidence has been found that there is a higher than expected concurrent incidence rate between them [4-9]. Of these published studies, the largest population-based study was conducted by Ojha et al. [5]. In this two-cohort group study, a bidirectional association was found between RCC and multiple myeloma. The first group were patients with RCC diagnosed as a primary malignancy, followed up for the incidence of PCM. The second group were patients with PCM diagnosed as a primary malignancy, followed up for the incidence of RCC. Both groups showed a higher than expected standardized incidence ratio (SIR); more specifically SIR = 1.51, 95% CI: 1.21-1.85 for the first group and SIR = 1.89, 95% CI: 1.47-2.40 for the second group.

Multiple hypotheses about the reasons for this association have been proposed. Some of them include treatment effects of the first cancer, age, lifestyle, environmental exposures, genetic mutations, and certain cytokines [3,5,6].

Given the eight-year history of previous treatment for PCM in our patient, treatment-induced RCC is a reasonable concern. Unfortunately, apart from lenalidomide, we do not have a precise history of the chemotherapeutic agents used for her previous treatment. We did not find any literature linking lenalidomide as a causative agent of RCC. In fact, it’s currently being investigated in phase I/II for RCC treatment [10]. Radiation therapy has rarely been linked to renal tumors after decades of exposure [11]. The effects of erythropoietin on the growth or suppression of RCC have been inconclusive [12]. Moreover, Ojha et al. [5] concluded that the bidirectional association found in their study aimed more towards shared risk factors, as seen in other similar associated diseases, rather than the treatment effect.

Increasing age is a common risk factor for both RCC and PCM. Our patient, at 68 years of age, is in the high-risk age bracket for both malignancies. However, Ojha et al. [5], showed in their study that the age-specific relative risk estimates were different for both cohort groups. The incidence of PCM after RCC had a bimodal distribution (ages 50-59 years and >80 years at highest risk), whereas the incidence of RCC after PCM was relatively consistent across the age groups examined. Consequently, they concluded that age alone may not be an explanation for the bidirectional association between these malignancies.

Obesity is a known risk factor for both RCC and PCM. Ojha et al. [5] hypothesize that, since adipose tissue is a major source of inflammatory mediators, such as interleukin-6, obesity could promote the development and growth of both neoplasms. Our patient was slightly overweight with a BMI of 25.4 at the time of admittance. Nonetheless, we do not have an available history of her weight prior to her chronic illness.

Interleukin-6 is produced by both RCC and PCM, which can support tumor growth and metastasis. In 1991, Sakai et al. [3] presented an interesting case of a 68-year-old male with concomitant PCM and RCC. They hypothesized that the interleukin-6 produced by the RCC could have stimulated the growth and proliferation of the PCM. Their hypothesis was supported by the fact that the bone marrow plasma cell involvement was significantly decreased after nephrectomy; specifically, from 20.5% to <5%.

In our patient, although it is not clear evidence, the intricate adherence of the peri-aortic PCM to the renal hilum at the area of the CCRCC, and the intimate and direct cellular involvement of these two malignancies, could possibly be due to cytokine interactions, as suggested by Sakai et al. [3], or, simply due to systemic PCM infiltration into renal parenchyma and CCRCC by chance or other unknown reasons. More studies are necessary to investigate a possible relationship. A close anatomical, yet not direct, relationship between these tumors was also described in the case series by Choueiri et al. [6] in which one of their cases presented with a large, 25.0 cm, renal plasmacytoma in the right kidney, and, a year later, developed a 4.0 cm, grade 2, CCRCC in the left kidney.

Most CCRCC is characterized by deletions and/or mutations on the short arm of chromosome 3 (3p) [2], but PCM has not been found to bear 3p abnormalities. In our review of literature, we did not find any common genetic abnormalities shared by common variant CCRCC and PCM.

## Conclusions

There is a clear and scientifically valid relationship between PCM and RCC demonstrated by literature. Although their simultaneous occurrence is a rare event, Ojha et al. demonstrated in their study that it occurs more often than predicted by chance alone. Multiple causes for this connection have been proposed which include, most emphatically, shared risk factors. Less studied proposed mechanisms are biochemical interactions between shared cytokines such as interleukin-6.

This case is the first to show a direct histologic relationship between both tumors, grossly and microscopically. The cause of this finding is not clear and could be due to chance alone, or other unknown reasons. This finding could further support the connection between these neoplasms demonstrated in the existing literature. Nevertheless, more studies are needed to further our understanding of the still obscure relationship between them.
